# Monoclonal antibody-based serological methods for detection of Cucumber green mottle mosaic virus

**DOI:** 10.1186/1743-422X-8-228

**Published:** 2011-05-15

**Authors:** Haili Shang, Yan Xie, Xueping Zhou, Yajuan Qian, Jianxiang Wu

**Affiliations:** 1 State Key Laboratory of Rice Biology, Institute of Biotechnology, Zhejiang University, Hangzhou, Zhejiang 310029, China

## Abstract

**Background:**

Cucumber green mottle mosaic virus (CGMMV), a member of the genus *Tobamovirus*, can be transmitted by seeds and infects many cucurbit species, causing serious yield losses in cucumber and watermelon plants. In this paper, five serological methods including antigen-coated plate enzyme-linked immunosorbent assay (ACP-ELISA), triple antibody sandwich enzyme-linked immunosorbent assay (TAS-ELISA), Dot-immunobinding assay (DBIA), direct tissue blot immunoassay (DTBIA) and immunocapture reverse transcriptase polymerase chain reaction (IC-RT-PCR) were described for detection and diagnosis of CGMMV.

**Results:**

Using the purified CGMMV particles as immunogens, six murine monoclonal antibodies (MAbs) were produced. Five serological methods were established using the MAb 4H1 and detection sensitivity was compared using purified preparations and infected-plant tissue extracts. The detection sensitivity of ACP-ELISA was 0.16 ng of purified CGMMV, whereas TAS-ELISA was more sensitive than ACP-ELISA with a minimum detection of 0.04 ng of purified CGMMV. The sensitivities of TAS-ELISA and DBIA were similar for detecting CGMMV in infected-plant tissue extracts, and were four times higher than ACP-ELISA. The IC-RT-PCR was the most sensitive method, which could detect as little as 0.1 pg of purified virus. The detection sensitivity of IC-RT-PCR for CGMMV-infected plant tissues was about 400 times higher than that of TAS-ELISA and DBIA.

**Conclusions:**

The established ACP-ELISA, TAS-ELISA, DBIA and DTBIA are suitable for routine CGMMV detection of large-scale samples in the field survey, while IC-RT-PCR is more sensitive and suitable for acquiring information about the viral genome.

## Background

Cucumber green mottle mosaic virus (CGMMV) is a species of the genus *Tobamovirus *and is an economically significant seed transmitted pathogen, which causes yield losses of about 15% in cucurbitaceous vegetable crops [1,2]. The virion of CGMMV is rod-shaped, approximately 300 nm in length and 18 nm in diameter [3]. CGMMV contains a single 6.4 kb plus-strand genomic RNA [4]. The most characteristic symptoms of the disease in cucurbit plants are systemic mosaic and mottling on leaves, and blistering and deterioration of fruit pulp [5]. CGMMV was first reported in the United Kingdom in 1935 [6]. Subsequently, it had been reported in Germany, Finland, Israel, Saudi Arabia, India, Pakistan, Korea and Japan [7-10]. To date, several isolates of CGMMV from Korea, Israel, Japan, Greece and Spain have been characterized based on serology and genomic sequences [1,4,11-15]. In 2003, a new disease with green mottle and mosaic symptoms occurred at watermelon and cucumber fields in northeast China [16]. In 2005, this disease developed an epidemic in watermelons in Liaoning province of China and caused considerable economic damage. The serological and reverse transcription-polymerase chain reaction (RT-PCR) detection results confirmed that the disease was caused by CGMMV [17]. CGMMV is an alien invasive pathogen [18] and it remains a potential serious threat to the production of cucurbitaceous crops in China.

A variety of techniques have been established for the detection and diagnosis of CGMMV: RT-PCR [4,15,19,20], real time RT-PCR [21], transmission electron microcopy (TEM) [1,22], immune capture (IC)-RT-PCR [11], ELISA using polyclonal antibodies (PAbs) [1,11,23] and monoclonal antibodies (MAbs) [2,5]. Among those detection methods, enzyme-linked immunosorbent assay (ELISA), Dot-immunobinding assay (DBIA) and direct tissue blot immunoassay (DTBIA) are more suitable for routine detection of large-scale samples in the field survey, while IC-RT-PCR is more sensitive and suitable for acquiring information about the viral genome [24]. In this study, six MAbs were produced and MAb-based ACP-ELISA, TAS-ELISA, DBIA, DTBIA and IC-RT-PCR methods for CGMMV detection were established.

## Materials and methods

### Virus sources and Virus purification

A CGMMV Liaoning isolate was kindly provided by Qing Chen (Xiamen Entry-Exit Inspection and Quarantine Bureau, Fujiang province, China) and used as antigens for raising PAbs and MAbs. The CGMMV isolate was maintained on *Cucumis sativus *cv. *Aohagauri *by mechanical inoculation in an insect-proof greenhouse. Tobacco mosaic virus (TMV), Odontoglossum ringspot virus (ORSV) and Tomato mosaic virus (ToMV) were characterized and maintained by author's laboratory.

Purified CGMMV particles were obtained from fresh infected leaf tissues as described by Zhou et al. [25]. The purified virions were mixed with 2% (w/v, g/mL) phosphotungstic acid (PTA) and examined with an electron microscope (JEM -1200 EX, JEOL Ltd., Tokyo, Japan)).

### Preparation of PAbs and MAbs against CGMMV

The purified CGMMV virions were used as an immunogen and PAbs against CGMMV were prepared in two New Zealand rabbits as described previously [26]. The rabbits were bled one week after the fifth injection, and the PAbs were used in TAS-ELISA.

Production of hybridomas secreting MAb against CGMMV was performed as described previously [26]. Hybridomas were injected intraperitoneally into pristane-primed syngeneic BALB/c mice to produce ascitic fluids. ACP-ELISA was used to determine the titres of ascitic fluids. MAb isotypes were determined by ELISA with the mouse MAb isotyping reagents according to the manufacturer's instruction (Sigma-Aldrich, St. Louis, MO, USA). Specificity analyses of MAbs and the purification of IgG were operated by the methods as described by Wu et al [27].

### ACP-ELISA and TAS-ELISA

Detection of CGMMV particles in purified preparations or in sap extractions of infected leaf tissues was carried out following the standard procedures for ACP-ELISA [28] and TAS-ELISA [27]. The working dilutions of the MAb and the goat anti-mouse IgG conjugated with alkaline phosphatase for ACP-ELISA were determined by phalanx tests. Briefly, the lane wells of ELISA plates coated samples were respectively added two-fold diluted MAb and incubated. The row wells of plates were respectively dispensed two-fold diluted the goat anti-mouse IgG conjugated with alkaline phosphatase and incubated. The alkaline phosphatase conjugate was detected with p-nitrophenyl phosphate. The working dilutions of the PAb and MAb for TAS-ELISA also were determined by phalanx tests. Briefly, the lane wells of ELISA plates were respectively coated two-fold diluted PAbs and incubated. After sample incubation, two-fold diluted MAbs were respectively dispensed in row wells of the ELISA plates and incubated. Goat anti-mouse IgG conjugated with alkaline phosphatase at 1:8000 dilution was subsequently applied into the wells and incubated. The alkaline phosphatase conjugate detection and the result analysis were performed as ACP-ELISA. Negative and positive controls were wells incubated with leaf extracts from healthy leaf and CGMMV-infected leaf tissues, respectively. All those samples were triturated in 0.01 mol L^-1 ^PBS buffer (pH 7.4) and two-fold diluted in the same buffer. The sample was considered to be positive when its absorbance value was three times greater than that of the negative control.

### DBIA and DTBIA

DBIA and DTBIA procedures were carried out according to the method described previously [29] and modified. Briefly, samples for DBIA were prepared by grinding leaf tissues in 0.01 mol L^-1 ^phosphate buffered saline (PBS) and centrifuged at 8000 ×g for 5 min. The tissue extracts were spotted on nitrocellulose membranes (Amersham Biosciences, Bucks, UK, 2 μL/spot) and allowed to be air-dry. The nitrocellulose membrane was soaked in 5% solution of dried skimmed milk in PBS for 30 min, followed by an incubation in a suitable dilution of MAb for 1 h. Nitrocellulose was rinsed four times in PBST (0.01 mol L^-1 ^PBS, 0.05% Tween-20, pH 7.4), then incubated in goat anti-mouse IgG conjugated with alkaline phosphatase (Sigma-Aldrich, St. Louis, MO, USA, 1:8000 in PBS) for another 1 h. After washing five times with PBST, the membrane was color-developed in a substrate solution, alkaline phosphatase buffer (0.1 mol L^-1 ^Tris base, 0.1 mol L^-1 ^NaCl and 0.05 mol L^-1 ^MgCl_2_, pH 9.5) containing NBT/BCIP (5-Bromo-4-Chloro-3-Indolyl phosphate/Nitro-Blue Tetrazolium chloride, Promega).

Tissue prints for DTBIA were prepared by transversely cutting young stems or rolled leaves with blades and gently pressed the freshly cut surface onto nitrocellulose membranes for 3 to 5 sec. The prints were air-dried and blocked for 30 min in 5% solution of dried skimmed milk in PBS. The ensuing steps for DTBIA were same as that of DBIA.

### IC-RT-PCR

The forward primer (CP-F: 5'-CTTACAATCCGATCACACCTAG-3') and the reverse primer (CP-R: 5'-CTAAGCTTTCGAGGTGGTAGC-3') used for IC-RT-PCR were designed based on the most conserved part of CGMMV *CP *gene obtained from GenBank, which were determined based on the alignment of CGMMV *CP *RNA sequences using the DNASTAR package (Version 7.0, DNAStar Inc., Madison, WI, USA). The IC-RT-PCR was performed as described previously [24]. Amplified DNA fragments were analyzed and sequenced as described previously [30].

## Results

### Virus purification

**Figure 1:**
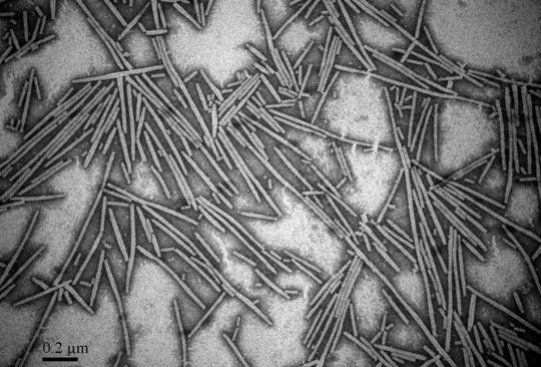
**Electron micrograph of purified cucumber green mottle mosaic virus**. Bar = 0.2 μm.

To produce antibodies against CGMMV, CGMMV particles were purified by differential centrifugation, and examined by transmission electron microscopy. Rod-shaped virions with about 300 nm in length and 18 nm in diameter were observed in the purified preparation (Figure 1), which was the typical morphology of virus particles in the genus *Tobamovirus*.

### Production and characterization of MAbs against CGMMV

Via cells fusion, cloning and antibodies detection, six hybridoma lines (4H1, 5B10, 5D11, 8E3, 11B12 and 11A4) secreting MAbs against CGMMV were obtained according to the method described by Köhler and Milstein [31], and each hybridoma was injected into pristine-primed BALB/c mice for producing ascitic fluid. The IgG yields of MAbs from ascitic fluids ranged from 6.53 to 16.72 mg mL^-1 ^(Table 1). The immunoglobulin classes and subclasses of 5B10, 8E3 and 11B12 were isotyped as IgG2a, while the other three MAbs (4H1, 5D11 and 11A4) were IgG1, and the light chains of all the six MAbs belonged to kappa chain (Table 1). The titers of those MAbs ranged from 10^-6^-10^-7 ^(Table 1).

**Table 1:** Properties of monoclonal antibodies to CGMMV

MAbs	Isotype	Ascites titre	IgG yield in ascites (mg/mL)
4H1	IgG1, κ chain	10^-7^*	12.98
5B10	IgG2a, κ chain	10^-6^	6.53
5D11	IgG1, κ chain	10^-7^	16.72
8E3	IgG2a, κ chain	10^-7^	10.02
11B12	IgG2a, κ chain	10^-7^	9.78
11A4	IgG1, κ chain	10^-7^	14.39

* The MAb titer was the last dilution that yielded an absorption value three times greater than that of the negative control.

**Figure 2:**
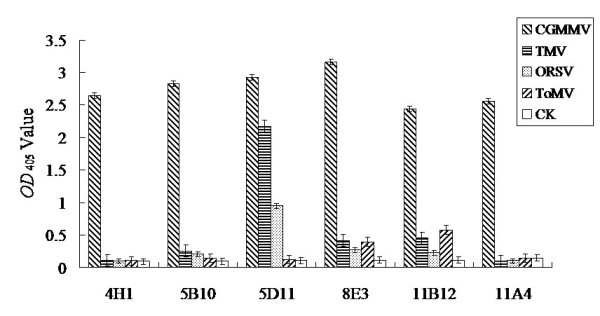
**Cross reactivities of anti-CGMMV MAbs with four tobamoviruses by ACP-ELISA**. The absorbance value was the mean value obtained from three independent assays at 30 min after adding the substrate at room temperature.

The cross reactivities of the MAbs with the other three tobamoviruses (TMV, ORSV and ToMV) were tested by ACP-ELISA and the results indicated that all six MAbs could strongly react with CGMMV in infected-plant tissue extracts, but not with healthy plants (Figure 2). MAbs 4H1, 5B10 and 11A4 reacted strongly only with CGMMV but not with the other three tobamoviruses, MAbs 8E3 and 11B12 reacted strongly with CGMMV and weakly with TMV and ToMV. MAb 5D11 reacted strongly with CGMMV, TMV and ORSV, but not with ToMV (Figure 2). Compared with other MAbs (5B10, 5D11, 8E3, 11B12 and 11A4), MAb 4H1 had the most sensitivity and specificity in the detection of CGMMV, which was therefore used further for assay development in this study.

### ACP-ELISA and TAS-ELISA for CGMMV detection

The working dilutions of MAb 4H1, PAb used as coating antibody in TAS-ELISA and goat anti-mouse IgG conjugated with alkaline phosphatase (Sigma-aldrich, St. Louis, MO, USA) were determined according to the results of phalanx tests. The results of the three repeated tests indicated that the dilution of MAb 4H1 at 1:5000, goat anti-mouse IgG conjugated with alkaline phosphatase at 1:8000 were suitable for ACP-ELISA, and the dilution of PAb at 1:5000, MAb 4H1 at 1:6000 and goat anti-mouse IgG conjugated with alkaline phosphatase at 1:8000 were suitable for TAS-ELISA.

**Figure 3:**
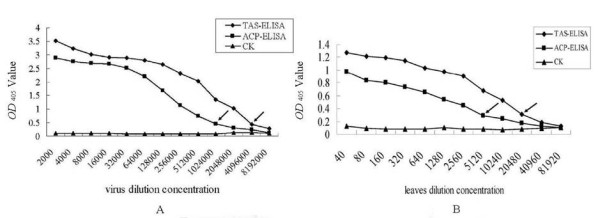
**Sensitivity analyses of ACP-ELISA (A) and TAS-ELISA (B)**. A: The sensitivities of ACP-ELISA and TAS-ELISA in the detection of purified CGMMV. The purified virus preparation was two-fold diluted in PBS buffer. CK: healthy tissues preparation was two-fold diluted in PBS buffer. The dilution endpoint of ACP-ELISA and TAS-ELISA were 1:1024000 and 4096000, corresponding to an equivalent of 0.16 ng and 0.04 ng of purified viruses respectively. B: The sensitivities of ACP-ELISA and TAS-ELISA in the detection of CGMMV in infected leaf extracts. CGMMV-infected leaf extracts were two-fold diluted in PBS buffer. CK: healthy leaf extracts were two-fold diluted in PBS buffer. The dilution endpoint of ACP-ELISA and TAS-ELISA were 1:5120 and 1:20480 (w/v, g mL^-1^), respectively.

Purified CGMMV and CGMMV-infected cucumber plants were used to determine the sensitivities of ACP-ELISA and TAS-ELISA. The results indicated that the detection limit of ACP-ELISA for purified viruses was 0.16 ng, while TAS-ELISA was more sensitive than ACP-ELISA and could detect 0.04 ng of purified viruses (Figure 3A). ACP-ELISA and TAS-ELISA could detect a minimum of CGMMV-infected leaf saps diluted at 1:5120 and 1:20480 (w/v, g mL^-1^), respectively (Figure 3B), which indicated that both methods were sensitive for the detection of CGMMV in field samples. The sensitivity of TAS-ELISA for purified virus preparation and viruses in infected leaf extracts was four times higher than that of ACP-ELISA.

### DBIA and DTBIA for CGMMV detection

**Figure 4:**
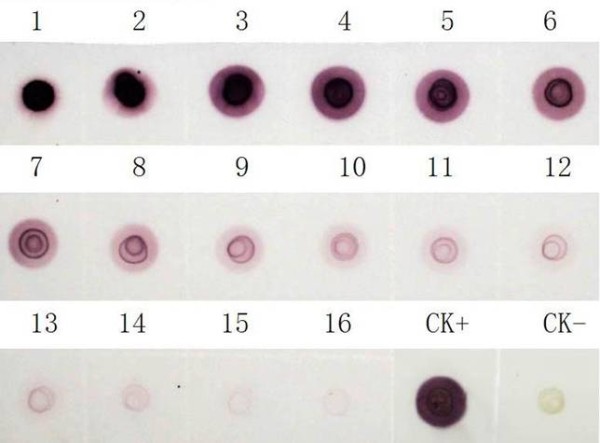
**Sensitivity analyses of DBIA for detecting CGMMV in infected plants**. 1-16, CGMMV-infected leaf tissue extracts two-fold diluted from 1:5 to 1:81920 (w/v, g mL^-1^) and the original concentration was 1 g mL^-1^. CK- and CK+, healthy and CGMMV-infected leaf tissue extracts diluted 1:30 (w/v, g mL^-1^), respectively. Brown spots indicate positive reaction and green spot indicates negative reaction.

DBIA and DTBIA for detecting CGMMV in infected plants were performed using nitrocellulose membranes as a sample support. The working dilutions of the MAb (4H1) and the enzyme-labelled second antibodies in DBIA and DTBIA procedures were chosen according to the results of phalanx tests as described above. The established DBIA could detect viruses in infected cucumber leaf tissue extracts diluted 1:20480 (w/v, g mL^-1^) and spots with virus were brown, while the visible spots of healthy (the negative control, CK-) diluted 1:30 (w/v, g mL^-1^) were weak green (Figure 4).

**Figure 5:**
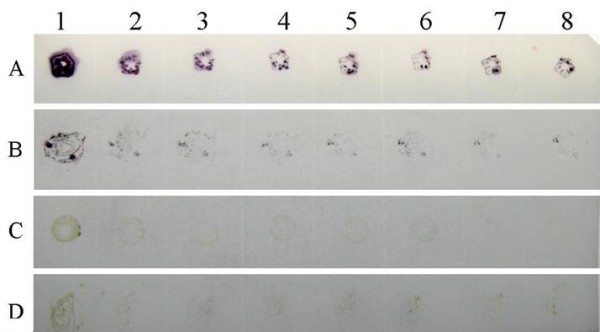
**Detection of CGMMV in infected cucumber plants by DTBIA**. The imprints from left to right were prepared from a single cut surface of same plant tissues. Lane A (1-8): Young stems infected with CGMMV; Lane B (1-8): Young leaves infected with CGMMV; Lane C (1-8): Healthy young stems; Lane D (1-8): Healthy young leaves.

In order to determine the suitable tissue for CGMMV detection by DTBIA, young stems and young fully expanded leaves of healthy or CGMMV-infected plants were sectioned and printed on nitrocellulose membranes. Brown-staining spots were observed in prints of young stems and leaves from CGMMV-infected plants, whereas green spots were observed in prints of young stems and leaves from healthy plants (Figure 5). The blots of fresh cut ends of young stems from CGMMV-infected plants shown stronger colour than that of young leaves, probably due to the rolled young leaves were less easy to handle as compared with young stems.

### IC-RT-PCR for CGMMV detection

**Figure 6:**
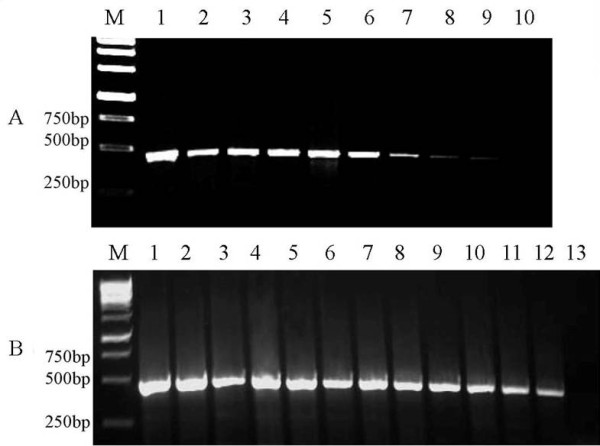
**Sensitivity of IC-RT-PCR for the detection of purified CGMMV (A) and CGMMV-infected plants (B)**. A: Purified virions with two-fold diluted from 1:5000 to 1:2560000 (from left to right). The original purified virus concentration was 1.28 μg mL^-1^. The lane 1 was diluted at 1:5000, corresponding to 256 pg of purified virus. The lane 9 was diluted at 1:1280000, corresponding to 0.1 pg of purified virus. B: CGMMV-infected leaf tissue saps with two-fold dilution from 1:200 to 1:819200 (w/v, g mL^-1^) (Lanes 1-11). Lane 12 was positive control and lane 13 was negative control. The original cucumber tissue extracts concentration was 0.05 g mL^-1^. M: 1 kb DNA marker.

IC-RT-PCR was successfully developed for the detection of purified CGMMV or CGMMV in infected plant extracts. The primers described above were designed for the amplification of a 480 bp fragment of the CGMMV *CP *gene. A 480 bp fragment was indeed amplified by IC-RT-PCR from both purified virus preparations (Figure 6A) and CGMMV-infected cucumber plant extracts (Figure 6B). The sensitivity of IC-RT-PCR for detecting purified CGMMV was at a minimum dilution of 1:1280000, corresponding to an equivalent of 0.1 pg of purified virus particles (Figure 6A, lane 9). For CGMMV-infected plant samples, the dilution endpoint was 1:102400 (Figure 6B, lane12).

The IC-RT-PCR amplified-fragments were cloned and sequenced. The sequences of clones were compared with the CGMMV *CP *sequences deposited in GenBank. The sequences of the PCR products had 97-99% homology with the *CP *region of the genome of CGMMV isolates in GenBank, Which confirmed that amplified products were derived from CGMMV *CP *gene.

## Discussion

Planting area of cucurbitaceous vegetable crops in China is over 3,000,000 hectare (ha), and is distributed in all provinces. The watermelon import and export trade among China, Japan and Korea is developing very quickly and might be the reason for the introduction of the virus into China. In 2005, an outbreak of a disease caused by CGMMV occurred in watermelon fields in Liaoning province, the damaged planting area was about 333 ha [17]. Methods for detection of CGMMV, an economically important seed transmitted virus, were not well established in China. In this study, five serological methods for CGMMV detection were established and a comparative analysis of these methods was assessed for their detection sensitivities of purified CGMMV and CGMMV-infected cucumber tissues. Both ACP-ELISA and TAS-ELISA could readily and specifically detect CGMMV. TAS-ELISA was more sensitive to detect CGMMV than ACP-ELISA. Both methods could be applied to detect CGMMV in filed samples.

The limit of detection by DBIA was similar to that of TAS-ELISA for CGMMV in infected-plant tissues. Short time and low costs are the main advantages of DBIA. DTBIA is a very convenient, specific and reliable method for detecting CGMMV under field conditions, and it can provide direct information about the distribution of the virus within host plants. So in a further detection application, plant samples can be spotted on nitrocellulose membranes at fields and be delivered to detect in local laboratories. Its simple and convenient advantage of this method is very significant implications for large-scale surveys as well as long-term epidemiological or ecological studies of this virus.

As expected, IC-RT-PCR is the most sensitive assay among the five methods and it could detect 0.1 pg of purified CGMMV. The sensitivity of this method for detecting CGMMV in infected-plant extracts was about 400 times higher than that of TAS-ELISA and DBIA. Moreover, the information of viral genome can be obtained from sequencing analyses of amplified products of IC-RT-PCR.

In conclusion, DTBIA is the most convenient method, while TAS-ELISA, ACP-ELISA and DBIA are also suitable for handing large amounts of samples in routine tests. Although IC-RT-PCR is not appropriate for large scale screening, it showed the best sensitivity than the other four methods, may be valuable for acquiring information about the viral genome of samples.

## Competing interests

The authors declare that they have no competing interests.

## Authors' contributions

HS had done most experiments and drafted the manuscript. YX had established the IC-RT-PCR and DTBIA methods for CGMMV detection. JW conceived of the study, and participated in its design and coordination. XZ, YQ and JW had proof-read and finalized the manuscript. All authors read and approved the final manuscript.

## References

[B1] Antignus Y Pearlsman M Ben YR Cohen S Occurrence of a variant of Cucumber green mottle mosaic virus in Israel Phytoparasitica 1990 18 50 56 10.1007/BF02980826

[B2] Antignus Y Wang Y Pearlsman M Lachman O Lavi N Gal-On A Biological and molecular characterization of a new cucurbit-infecting tobamovirus Phytopathology 2001 91 565 571 10.1094/PHYTO.2001.91.6.565 18943945

[B3] Tan SH Nishiguchi M Murata M Motoyoshi F The genome structure of kyuri green mottle mosaic tobamovirus and its comparison with that of cucumber green mottle mosaic tobamovirus Arch Virol 2000 145 1067 1079 10.1007/s007050070110 10948983

[B4] Ugaki U Tomiyama M Kakutani T Hidaka S Kiguchi T Nagata R Sato T Motoyoshi F Nishiguchi M The complete nucleotide sequence of cucumber green mottle mosaic virus (SH strain) genomic RNA J Gen Virol 1991 72 1487 1495 10.1099/0022-1317-72-7-1487 1856687

[B5] Shim CK Lee JH Hong SM Han KS Kim HK Construction of antibodies for detection and diagnosis of Cucumber green mottle mosaic virus from watermelon plants Plant Pathol J 2006 22 21 27

[B6] Ainworth GC Mosaic disease of cucumber Ann Appl Biol 1935 22 55 67 10.1111/j.1744-7348.1935.tb07708.x

[B7] Komuro Y Tochihara H Fukatsu R Nagai Y Yoneyama S Cucumber green mottle mosaic virus on watermelon in Chiba and Ibaraki Prefectures (in Japanese) Ann Phytopathol Soc Jpn 1968 34 377

[B8] Francki RIB Hu J Palukaitis P Taxonomy of cucurbit infecting Tobamovirus as determined by serological and molecular hybridization analyses Intervirology 1986 26 156 163 10.1159/000149695 3583663

[B9] Lee KW Lee BC Park HC Lee YS Occurrence of cucumber green mottle mosaic virus disease of watermelon in Korea Korean J Plant Pathol 1990 6 250 255

[B10] Lee KY Current occurrence and control of CGMMV 'Konjak' disease Plant Dis Agric 1996 2 28 39

[B11] Celix A Luis-Arteaga M Rodriguez-Cerezo E Frist report of Cucumber green mottle mosaic Tobamovirus infecting greenhouse-grown cucumber in Spain Plant Dis 1996 80 1303

[B12] Varveri C Vassilakos N Bem F Characterization and detection of Cucumber greem mottle mosaic virus in Greece Phytoparasitica 2002 5 493 501

[B13] Kim SM Lee JM Yim KO Oh MH Park JW Kim KH Nucleotide sequences of two Korean isolates of Cucumber green mottle mosaic virus Mol Cell 2003 16 407 412 14744034

[B14] Ko SJ Lee YH Cha KH Lee SH Choi HS Choi YS Lim GC Kim KH Incidence and distribution of virus disease on cucumber in Jeonnam procince during 1999-2002 Plant Pathol J 2006 22 147 151

[B15] Shim CK Han KS Lee JH Bae DW Kim DK Kim HK Isolation and charatracterization of watermelon isolate of cucumber green mottle mosaic virus (CGMMV-HY1) from watermelon plant with severe mottle mosaic symptoms Plant Pathol J 2005 21 167 171

[B16] Qin BX Cai JH Liu ZM Chen YH Zhu GN Huang FX Preliminary identification of a Cucumber green mottle mosaic virus infecting pumpkin (in Chinese with abstract in English) Plant Quarantine 2005 4 198 200

[B17] Chen HY Zhao WJ Cheng Y Li MF Zhu SF Molecular identification of the virus causing watermelon mosaic disease in Mid-Liaoning Acta Phytopatho Sin 2006 4 306 309

[B18] Chen J Li MF An alien invasive pest-cucumber green mottle mosaic virus (in Chinese with abstract in English) Plant Quarantine 2007 2 94 96

[B19] Yoon JY Choi GS Choi SK Hong JS Choi JK Kim W Lee GP Ryu KH Molecular and biological diversities of Cucumber green mottle mosaic virus from cucurbitaceous crops in Korea J Phytopahol 2008 156 408 412 10.1111/j.1439-0434.2007.01376.x

[B20] Liu Y Wang YN Wang XF Zhou GG Molecular characterization and distribution of Cucumber green mottle mosaic virus in China J Phytopahol 2009 157 393 399 10.1111/j.1439-0434.2008.01509.x

[B21] Chen HY Zhao WJ Gu QS Chen Q Lin SM Zhu SF Real time TaqMan RT-PCR assay for the detection of cucumber green mottle mosaic virus J Virol Methods 2008 149 326 329 10.1016/j.jviromet.2008.02.006 18359519

[B22] Ibrahim M Al SW Omer AA A strain of Cucumber green mottle mosaic virus (CGMMV) from Bottlegourd in Saudi Arabia J Phytopathology 1992 134 152 156 10.1111/j.1439-0434.1992.tb01223.x

[B23] Mitsuhiro S Takayoshi O Yoshiteru S A new source of Resistance to Cucumber green mottle mosaic virus in melon J Japan Soc Hort Sci 2006 75 469 475 10.2503/jjshs.75.469

[B24] Wu JX Meng CM Shang HL Rong S Zhang C Hong J Zhou XP Monoclonal antibody-based triple antibody sandwich-enzyme-linked immunosorbent assay and immunocapture reverse transcription-polymerase chain reaction for odontoglossum ringspot virus detection J Virol Methods 2011 171 40 45 10.1016/j.jviromet.2010.09.027 20933014

[B25] Zhou XP Chen JS Li DB Li WM A method of purification of potyviruses with high yield Chinese Microbiol 1994 21 184 186

[B26] Wu JX Yu C Yang CY Deng FL Zhou XP Monoclonal antibodies against the recombinant nucleocapsid protein of tomato spotted wilt virus and its application in the virus detection J Phytopahol 2009 157 344 349 10.1111/j.1439-0434.2008.01498.x

[B27] Wu JX Yu L Li L Hu JQ Zhou JY Zhou XP Oral immunization with transgenic rice seeds expressing VP2 protein of infectious bursal disease virus induces protective immune responses in chickens Plant Biotechnol J 2007 5 570 578 10.1111/j.1467-7652.2007.00270.x 17561926

[B28] Wu JX Meng CM Shang HL Rong S Zhang C Hong J Zhou XP Monoclonal antibody-based triple antibody sandwich-enzyme-linked immunosorbent assay and immunocapture reverse transcription-polymerase chain reaction for Odontoglossum ringspot virus detection J Virol Methods 2011 171 40 45 10.1016/j.jviromet.2010.09.027 20933014

[B29] Njukeng AP Atiri GI Hughes JDA Winter SS Development of serological procedures for rapid, sensitive and reliable detection of Yam Mosaic Virus genus Potyvirus in yam tissues Trol Sci 2004 44 136 147 10.1002/ts.155

[B30] Jiang JX Zhou XP Maize dwarf mosaic disease in different regions of China is caused by Sugarcane mosaic virus Arch Virol 2002 147 437 2443 10.1007/s705-002-8332-5 12491109

[B31] Köhler G Howe SC Milstein C Fusion between immuno globulin-secreting and nonsecreting myeloma cell lines Eur J Immunol 1976 6 292 295 10.1002/eji.1830060411 825374

